# Minimally Invasive Plating of Distal Radius Fracture: A Series of 42 Cases and Review of Current Literature

**DOI:** 10.1155/2023/3534849

**Published:** 2023-02-23

**Authors:** Oryza Satria, Rio Wikanjaya, Christa Adriane Tenges, Muslich Idris Al Mashur

**Affiliations:** ^1^Department of Orthopaedic and Traumatology, Fatmawati Central General Hospital, Jakarta 12430, Indonesia; ^2^Department of Orthopaedic and Traumatology, Dr. Cipto Mangunkusumo Hospital, Faculty of Medicine Universitas Indonesia, Jakarta 10430, Indonesia; ^3^Department of Physical Medicine and Rehabilitation, Fatmawati Central General Hospital, Jakarta 12430, Indonesia

## Abstract

Surgical techniques developed for distal radius fracture fixation have become increasingly advanced, including minimally invasive plate osteosynthesis (MIPO). This study aimed to introduce and evaluate the functional outcome of a novel MIPO technique that differs from previous reports. This study included 42 patients with distal radius fractures who underwent minimally invasive surgical plating of the distal radius. All patients were treated with closed reduction, fixation using K-wire, and subsequent insertion of a volar anatomical stable angle short plate on the distal radius. An arthroscopy-assisted evaluation and repair procedure were performed to correct intra-articular involvement, triangular fibrocartilage complex tears, and scapholunate injuries. Functional outcomes were assessed using a visual analog scale score; quick disabilities of the arm, shoulder, and hand score; and postoperative range of motion of flexion, extension, supination, and pronation at the 3-month follow-up, showing significant improvement in all parameters (all *p* ≤ 0.05). This study provides a simpler yet reliable method with reproducible and consistent results to treat distal radius fractures using minimally invasive plating with closed reduction and plate insertion, resulting in satisfactory clinical outcomes in all patients.

## 1. Introduction

Distal radius fractures have been recognized as the most evolving treatment with a significant milestone. They are one of the most common orthopedic injuries, accounting for 8%–15% of all bone injuries found in the adult and elderly population. Their occurrence in humans has been recorded as early as 5000 years ago in an ancient Egyptian case report within the *Edwin Smith Papyrus* [1]. Before the invention of röentgenography, Pouteau (1783), Colles (1814), and Dupuytren (1847) described distal radius fractures and how to reduce them, including the importance of immobilization. While further discussion regarding the degree of displacement and articular involvement has become an emerging issue since the invention of roentgenography, treatment was still nonoperative using casts or traction [1, 2].

Operative treatment was reported for the first time in 1908 when Lambotte operated on a distal radius fracture using percutaneous K-wires through radial styloid to maintain reduction. However, intraarticular comminution still became a significant issue. Anderson and O'Neil addressed this issue using the ligamentotaxis concept with a joint-spanning external fixator. A significant advancement was made when a group of Swiss surgeons met to discuss fracture management, known as *Arbeitsgemeinschaft für Osteosynthesefragen* (AO). Shortly afterward, two case reports were published due to growing interest in operative management of distal radius fractures. Since then, the operative treatment of distal radius fractures has grown rapidly, including volar and dorsal plating, distraction plating, intramedullary implants, and the arthroscopic approach [1, 2]. While operative approaches have been used for complex distal radius fractures, there are potential complications, including loss of fixation, reflex sympathetic dystrophy, neuritis, and wound infection. Minimally invasive plate osteosynthesis (MIPO) of a distal radius fracture offers a solution to overcome these complications [3, 4].

Recent literature regarding the MIPO approach for distal radius fractures has been reported by Liverneaux [5] aiming to overcome potential complications from open surgery. MIPO aims to decrease skin incisions and preserve the periosteum and pronator quadratus. Preservation of the associated anatomical parts may favor bone healing. In addition, a minimally invasive approach would increase patient satisfaction with a pleasant cosmetic appearance. This case series reports a novel minimally invasive technique, including closed reduction and smaller plate insertion, and its outcome.

## 2. Materials and Methods

### 2.1. Patient Selection

This study included 42 patients with distal radius fractures who underwent minimally invasive surgery plating of the distal radius between January 2021 and December 2021. This study was approved by our institution's Ethics Committee (approval number: UM.01.05/VIII.5/412/2022), and informed consent was obtained from all patients. We included patients with AO/Orthopaedic Trauma Association type A2, A3, or B3 distal radial fractures for MIPO. Type A2 was an extra-articular radius fracture with an impacted fragment. Type A3 was an extraarticular radius fracture involving multiple fragments. Type B3 was a partial articular radius fracture, with the primary fracture in the coronal plane involving the palmar rim.

### 2.2. Operative Procedure

**Figure 1:**
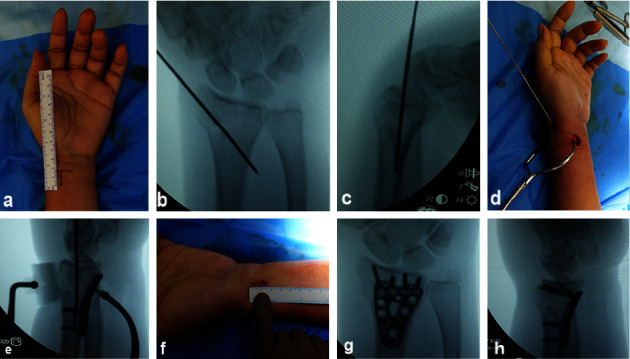
Minimally invasive plating using the extra-articular technique in Case 1: (a) incision design, (b, c) closed reduction and provisional fixation using K‐wire, (d, e) soft tissue-bone reduction forceps were used to grip the plate over the bone, (f) the 15 mm wound was closed with an absorbable suture, and (g, h) the final result viewed under an image intensifier.

**Figure 2:**
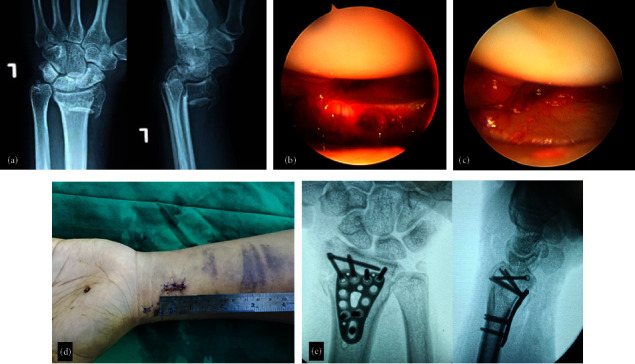
Arthroscopy-assisted intraarticular fracture reduction in Case 2: (a) preoperative X‐ray, (b, c) reduction using arthroscopy, (d) closed wound, and (e) final result.

The operating procedure was adopted from Livernaux [5] with simplifying modifications. All patients were operated on under general anesthesia using the same techniques by a single surgeon (OS). An “ExtremiLock™ wrist short narrow plate (Osteomed, USA) was used in this series. Technically, “ExtremiLock™ is easier to use due to its smaller and shorter size. The patient was positioned supine with the affected arm on a Mayo table. Various techniques were performed closed to achieve acceptable reduction under an image intensifier. This step was essential because the fracture's reducibility determined the next step.  Figure 1 shows various techniques in closed reduction. After achieving an acceptable reduction, one or two 1.4 mm Kirschner wires were introduced obliquely from the radius styloid process to the proximal ulnar side as a provisional fixation. Then, a 1.5 cm incision was made using the flexor carpi radialis (FCR) approach, 2 cm proximal to the tip of the radial styloid. The flexor carpi radialis sheath was also dissected longitudinally to the same extent. Except for the radial artery, all tendons, muscles, and nerve structures were ulnarly retracted until the pronator quadratus was exposed. A specialized periosteal elevator was used to elevate the pronator quadratus proximally to create space for the site of the plate. Then, the plate was inserted beneath the elevated pronator quadratus over the reduced fracture, and fixation was completed using locking screws. Care should be taken to not violate the radiocarpal joint using fluoroscopy guidance. The operative procedure is shown in Figures 1 and 2.

Outcomes were assessed by comparing preoperative and postoperative evaluations. Postoperative follow-up included pain measurement on the visual analog scale (VAS) from 0 (no pain) to 10 (maximum pain imaginable). Global hand function was evaluated using the quick disabilities of the arm, shoulder, and hand (DASH) score from 0 (normal upper limb function). Wrist mobility was measured in flexion, extension, pronation, and supination. Radiological criteria included volar tilt, radial inclinations, and ulnar variance.

### 2.3. Statistical Analysis

Univariate analyses were performed to describe the data distribution and measure the mean and standard deviation of symmetric distributions and the median and interquartile range of asymmetric distributions. Bivariate analyses using paired *t*-tests for symmetric and Wilcoxon signed-rank tests for asymmetric distributions were used to determine the relationship between each variable. All results with *p* ≤ 0.05 were considered statistically significant. The collected data were analyzed using the SPSS v.26 software. The same study authors performed all statistical analyses.

## 3. Results

### 3.1. Subject Characteristics

This study included 42 patients with distal radius fractures who underwent minimally invasive surgery, of which 24 were male (57.1%) and 18 were female (42.9%) with a mean age of 41.14 years (range 21–65). The patients' demographic, clinical, and radiological data are shown in Table 1.

**Figure 3:**
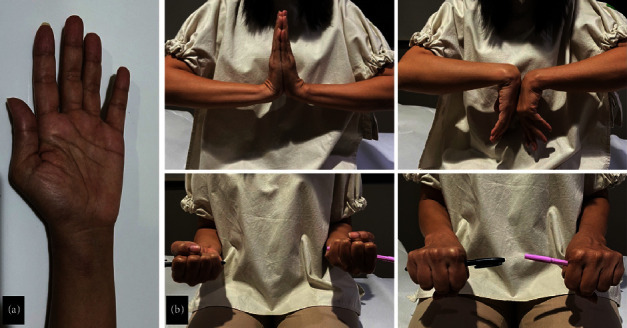
Clinical examination after six months of postoperative follow-up in case 1.

**Figure 4:**
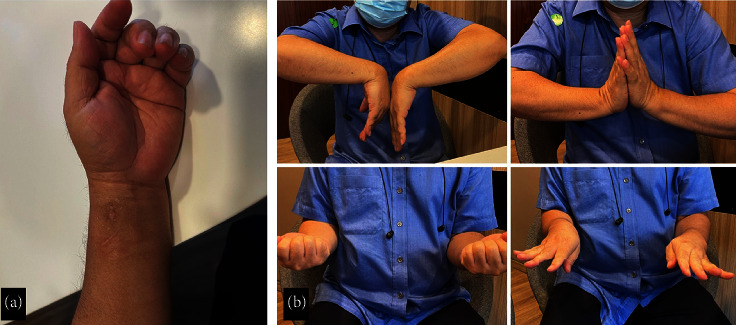
Clinical examination after six months of postoperative follow-up in case 2.

All patients underwent a minimally invasive approach using closed reduction and plate and screw fixation. Arthroscopy was performed in six patients with intraarticular involvement. Detailed information on each patient's fracture is provided in Table 2. Their postoperative six-week follow-up is shown in Figures 3 and 4. Two patients reported early complications, such as stiffness and wound breakdown. However, the complications resolved on their own.

### 3.2. Postoperative Outcomes

We found significant differences in VAS score, functional outcome, and range of motion (ROM) between postoperative and three-month follow-ups (Table 3). Clinical examination of the patients at their three-month follow-up is shown in Figure 3.

## 4. Discussion

Our study aimed to evaluate a minimally invasive technique that includes closed reduction and plate insertion in patients with distal radius fractures. It included 42 patients with a mean age of 41.14 years (range 21–65). A large epidemiological study by Azad et al. examining 1,124,060 distal radius fractures showed that they occurred most frequently in the pediatric (10–14 years) and elderly (>65 years) populations. The contrasting ages of our patients reflect our study's much smaller cohort [6]. However, previous distal radius reports specifically discuss MIPO's relatively similar demographic population [4, 7, 8].

The variation in fracture type was also present in our patients. In addition, some also had associated lesions, such as triangular fibrocartilage complex (TFCC) tears and scapholunate injuries. A previous study by Wei et al. included different fracture classifications, including type A2, A3, B1, B3, C1, and C2. This variation is important because heterogenous subjects may affect the study outcome. However, we performed various reduction techniques to overcome such variation and obtain optimal articular congruency in all cases. Associated injuries, including TFCC tears and intraarticular involvement, were treated using arthroscopy-assisted TFCC repair. This arthroscopy-assisted procedure in MIPO of the intraarticular distal radius is required to resolve intraarticular congruency and associated intraarticular injury and has been performed in many previous reports [4, 7].

Several previous reports have been published on conventional and minimally invasive techniques for distal radius fractures [9, 10]. The conventional technique requires an incision in the skin and pronator quadratus muscle with a further detachment of the periosteum in the fracture area. The MIPO technique was proposed due to its better fracture healing and cosmetic outcomes. In addition, a minimally invasive technique generally preserves blood supply adjacent to the pronator quadratus muscle, resulting in less muscular damage.

Swiontkowski et al. [9] compared conventional and MIPO techniques, using brachial plexus block as the primary MIPO anesthesia. Lebailly et al. [7] also used regional anesthesia in all distal radius fracture patients treated with a minimally invasive technique. Peripheral nerve blocks such as brachial plexus have been associated with reduced opioid doses and shorter hospital stays. Therefore, they are frequently used in upper extremity surgeries, such as those for distal radius fractures [11].

However, our study used general anesthesia. Romero Prieto et al. [12] previously reported using general anesthesia to treat distal radius fractures. General anesthesia may be suitable for patients with anxiety, long surgery duration, or contraindications for regional anesthesia. General anesthesia is also safe and economical despite several potential drawbacks compared to peripheral nerve block [11].

The MIPO procedure proposed by Swiontkowski et al. [9] was similar to ours, involving closed reduction with subsequent intrafocal pinning with the Kapandji technique. The incision was made transversely along the transverse wrist skin crease. Zemirline et al. also used external manipulation for reduction with fixation using K-wires. However, they also used arthroscopy for high-energy trauma or intraarticular involvement. Despite the same incisional approach in both studies, Zenke et al. used a longer single transverse incision (3 cm vs. 1.5 cm). Both studies showed no significant difference in overall outcomes between conventional and MIPO procedures [8]. This result was also supported by our findings, where an average 1.5 cm transverse incision was sufficient without complications such as secondary displacement postoperatively and after follow-up.

The shorter incision is reasonable while using a smaller plate for fixation. In contrast, Pire et al. [10] used Henry's approach, which uses a 15 mm proximal longitudinal incision to the radial styloid apex. They used a long volar plate under the pronator quadratus. They reported better cosmetic and economic advantages with MIPO compared to the conventional procedure, also without significant differences in clinical outcomes [10]. Wei et al. also used a longitudinal incision, although they used two rather than one. They also reported overall satisfactory clinical outcomes in their patients [4]. Their rationale for using a longitudinal incision was to allow sufficient access to the radial epiphysis and better cosmetic results than a transversal incision. Transverse incisions have been reported to be bothersome for patients, usually misinterpreted as a phlebotomy scar. There was also a higher risk for iatrogenic injury related to persistent pain in a transverse incision [9, 13].

The most similar technique to ours was that of Liverneaux et al. However, there are several differences. They used regional rather than general anesthesia and performed reduction prior to surgery. In addition, our study used a smaller plate to allow the use of shorter incisions. Liverneaux also used several maneuvers to reduce the fracture by open means, including the “tyre changer maneuver,” in which a periosteal elevator was inserted into the fracture site's volar aspect; the “dorsal leverage maneuver,” in which percutaneous K-wire was inserted into the fracture site's radial aspect, and the “compression maneuver,” in which a compression screw passed through the oblong hole [5].

Our study preferred closed reduction using various maneuvers. After reduction, K-wires were inserted to provisionally maintain the reduction. Then, the plate was applied to the radius's anterior cortex for fixation. Arthroscopy was used for intraarticular involvement. The procedure we performed was analogous to internal fixation using cephalomedullary nailing, resulting in the advantages of good soft-tissue preservation, radiographically measurable reduction, and a relatively short elapsed time from incision to wound closure. Biz et al. also used additional K-wires, finding them associated with better clinical outcomes in the elderly and younger adults. Additional K-wires may provide additional stability to the fracture area. However, they did not use plates as the main fixator to the fracture [14].

It should also be noted that Liverneaux used a lift-off technique, which cannot be used for comminuted fractures, fractures with relatively thin and distally located fragments, and osteoporotic lesions. This technique was required because the procedure that Liverneaux proposed used strength/integrity from the distal part. Therefore, this procedure may lead to pull-out in cases where distal integrity is absent.

It must be noted that using smaller and shorter plates for MIPO in distal radius fracture cases has not been previously reported. Based on our experience, using smaller and shorter plates had several advantages, such as being easier to perform and safer for adjacent soft tissues. Wrong plate size may impede adequate bone healing and lead to displacement after follow-up. However, the fracture remained stabilized, and no complications, such as secondary displacement, occurred. Therefore, we recommend using smaller and shorter plates in MIPO to make the procedure easier and to optimize patient outcomes.

Outcome measurements indicated satisfaction in our patients. It should be noted that ROM and VAS significantly improved between the postoperative and three-month follow-ups (*p* < 0.05). Subjective measurements using the DASH score also indicated improvement at three months postoperative (*p* < 0.05). A similar result was observed in postoperative VAS scores, with the procedure resulting in less postoperative pain. Final clinical outcomes were comparable to preinjury conditions, with no clinical complaints after three months of follow-up. A previous study of 144 distal radius fracture cases by Lebailly et al. [7] also reported a mean pain score of 1.8, an average quick-DASH score of 25, a ROM of >85%, and no prescribed postoperative physiotherapy in all patients treated with a minimally invasive approach. Moreover, we found no major complications in any of our patients. This observation suggests that MIPO effectively manages distal radius fractures, whether a closed or open reduction was performed compared to conventional open reduction and internal fixation.

A minimally invasive approach also effectively treated distal radius fractures with long-segment metadiaphyseal comminution. Wei et al. used percutaneous MIPO in nine patients, all of whom reported complete healing and excellent wrist function based on DASH score, ROM, and grip strength [15]. In high-energy trauma or articular involvement, arthroscopy is preferred to reduce osteochondral fragments and repair scapholunate lesions after plate fixation. A similar procedure was performed by Zemirline et al., who also showed satisfactory functional outcomes using the DASH score. The advantage of using arthroscopy was that it allows better reduction and assessment of lesions adjacent to the fracture. They used an incision of 15 mm in the anterior, the same as in our cases. Smaller incisions are associated with aesthetic scars and better patient satisfaction [8].

A major drawback of this procedure is radiation exposure due to fluoroscopy, which produces harmful ionizing radiation. Radiation effects can be divided into stochastic and deterministic types [16]. Stochastic effects have no threshold dose, and as exposure increases, the possibility (rather than the intensity) of the effect increases. There are long-term consequences, such as genetic damage and cancer, caused by radiation. Deterministic effects are dosage dependent and require a minimum threshold dose to manifest. Below that dose, no effect occurs. Skin damage is the most common deterministic adverse effect of fluoroscopy radiation exposure. Furthermore, the steep learning curve and technical difficulties may hinder this technique's development. Consequently, reducing radiation exposure and the learning curve is important for the patient's procedure success and surgical outcome.

This case series also had limitations. It included various fracture types, which may affect surgical outcomes. Some fractures had intraarticular involvement, which was addressed using arthroscopy. Some patients also had simultaneous TFCC injuries, which required repair. Furthermore, since this study only had a small subject size, its findings are not conclusive. Despite its heterogeneity, the modified MIPO proposed by our study resulted in comparable functional outcomes with shorter surgery times. A prospective study with the same baseline characteristics may provide more information with less bias.

## 5. Conclusions

MIPO in managing distal radius fractures was associated with satisfactory clinical outcomes and reproducible and consistent results. Smaller plates provided a simpler but reliable method with no reported complications after the follow-up period. However, further studies with homogenous fracture types and longer follow-up periods are needed to confirm our findings.

## Figures and Tables

**Table 1:** Subjects' demographic, clinical, and radiographic characteristics.

	*n* = 42
Men	24 (57.1%)
Women	18 (42.9%)
Age	41.14 ± 11.65
AO-OTA	
23-A2	18 (42.9%)
23-A3	16 (38.1%)
23-B3	8 (19.0%)
Incision	15.00 (12–18) cm
Tourniquet time	71.86 ± 24.17 min
Volar tilt	7.50° ± 2.62°
Radial inclination	13.36° ± 5.11°
Ulnar variance	−1.00° (-2°–2°)

**Table 2:** Subjects' fracture characteristics.

No.	Sex	Age	Dominant side	Affected side	Classification AO	Associated injury	Arthroscopy			Complication
1	M	34	R	R	23-A3					
2	M	23	R	L	23-A2					
3	M	32	R	L	23-B3	TFCC tear		Yes		Stiffness
4	M	34	R	R	23-A3					
5	F	56	R	R	23-A3					
6	F	54	R	L	23-A2					
7	F	34	R	L	23-A2					
8	F	45	R	R	23-A2					
9	M	47	R	L	23-B3	Scapholunate injury		Yes		
10	M	34	R	L	23-A3					
11	M	22	R	R	23-A3					
12	F	21	R	L	23-A3					Wound breakdown
13	M	23	R	R	23-A2					
14	M	45	R	L	23-A3					
15	M	23	R	R	23-B3					
16	F	34	R	L	23-A2					
17	F	60	R	R	23-A3					
18	F	65	R	L	23-A2					
19	F	47	R	L	23-A2					
20	F	56	R	R	23-B3					
21	M	34	R	R	23-A2					
22	M	56	R	L	23-B3	TFCC tear + scapholunate injury			Yes	
23	M	54	R	L	23-A3					
24	M	56	L	R	23-A3					
25	M	47	R	R	23-A2					
26	F	34	R	R	23-A2					
27	M	32	R	L	23-A3					
28	M	27	R	L	23-A2					
29	M	54	R	R	23-A3					
30	F	35	R	L	23-A2					
31	F	36	R	L	23-B3	TFCC tear		Yes		
32	F	37	R	R	23-A3					
33	M	41	R	R	23-A3					
34	F	43	R	L	23-B3			Yes		
35	M	44	R	L	23-A3	TFCC tear				
36	F	57	L	R	23-A2					
37	F	42	R	R	23-A2					
38	M	33	R	R	23-A2					
39	M	38	R	L	23-A3					
40	M	56	R	L	23-B3	TFCC tear		Yes		
41	F	43	R	R	23-A2					
42	M	40	R	L	23-A2					

**Table 3:** Surgical outcomes of subjects treated with MIPO at their postoperative and 3-month follow-ups.

Parameters	Postoperative	3-month follow-ups	*p* value
VAS score	1.00 (0–2)	1.00 (0–2)	0.016
Quick-DASH score	33 (12–60)	29 (11–59)	0.0001
ROM-flexion	69.17 ± 12.95	74.88 ± 11.336	0.0001
ROM-extension	74.6 (34–97)	81.24 (45–100)	0.0007
ROM-pronation	75.46 ± 10.05	83.02 ± 9.78	0.0001
ROM-supination	73.34 ± 11.54	83.83 ± 11.59	0.0001

## Data Availability

The patient's detailed data used to support the findings of this study are available from the corresponding author upon request.
